# Is L-PRF an effective hemostatic agent in single tooth extractions? A cohort study on VKA and DOAC patients

**DOI:** 10.1007/s00784-023-04880-z

**Published:** 2023-01-28

**Authors:** Federico Berton, Fulvia Costantinides, Claudio Stacchi, Ambra Corradini, Andrea Di Lenarda, Roberto Di Lenarda

**Affiliations:** 1 Maxillofacial and Dental Surgical Clinic, Department of Medical, Surgical and Health Sciences, University of Trieste, Trieste, Italy; 2 Cardiovascular Center, University Hospital and Health Services, Trieste, Italy

**Keywords:** PRF, Direct oral anticoagulants, DOAC, Novel oral anticoagulants, NOAC, Tooth extraction, Bleeding risk

## Abstract

**Objectives:**

The aim of this clinical observational study was to assess the efficacy of L-PRF as a hemostatic agent in patients under treatment with vitamin K antagonists (VKAs) or direct oral anticoagulants (DOACs).

**Materials and methods:**

Patients under oral anticoagulant therapy (VKA or DOACs) who needed a single simple tooth extraction were enrolled. L-PRF plug was positioned inside the alveolus and secured with non-absorbable sutures. Surgical time, pain-VAS, paracetamol intake, intra-operative, post-operative biological complications, and bleeding events have been registered.

**Results:**

A total of 112 patients (59 patients for DOAC and 53 for VKA group) were enrolled. Post-operative bleeding was recorded in nine patients (17%) for VKA group and nine patients (15.3%) for DOACs group. None of the patients needed a medical support for managing of bleeding. Seven days after surgery, no cases of post-extractive complications occurred.

**Conclusions:**

The use of L-PRF resulted in limited mild late post-operative bleedings without the need of medical intervention.

**Clinical relevance:**

The use of L-PRF can be adopted for an uneventful post-operative curse in anticoagulated patients without chasing their therapy for single tooth extraction.

**Supplementary Information:**

The online version contains supplementary material available at 10.1007/s00784-023-04880-z.

## Introduction

Due to the increase in average life expectancy in recent decades, we are witnessing an evident increase of elderly population and, in the same time, a significant increase in the prevalence of chronic pathologies such as cardiovascular diseases [1].

Frequently reported cardiovascular pathological conditions, such as non-valvular atrial fibrillation, valvular pathologies, myocardial infarction, stroke, pulmonary embolism, and deep vein thrombosis, require oral anticoagulant therapy as prophylaxis or *quoad vitam* pharmacological treatment [2]. The purpose of anticoagulation therapy is to reduce blood clotting capacity in order to reduce the risk of thromboembolic complications in clinical conditions such as atrial fibrillation, mechanical heart valves, deep vein thrombosis and pulmonary embolism, and stroke.

Although traditional, oral anticoagulant therapy with vitamin K antagonists (VKAs), warfarin and acenocoumarin, has represented the gold standard for decades; nowadays, this drug category has been largely supplanted by direct oral anticoagulants, briefly named DOACs.

The introduction and development of DOACs was dictated by the need to overcome the several disadvantages associated with traditional oral anticoagulant therapy, such as the interactions with food and other medicaments, the long half-life, the need of individual dosages, and the mandatory regular monitoring.

DOACs include dabigatran etexilate (Pradaxa) which acts as a direct inhibitor of thrombin or factor IIa, rivaroxaban (Xarelto), apixaban (Eliquis), and edoxaban (Lixiana) which blocks blood clotting factor Xa [3].

Sparse clinical studies are present, to date, in literature regarding the management of DOAC patients in oral surgery, as DOACs are new generation drugs: this forces healthcare professionals to operate following protocols supported by scarce scientific evidence [4].

Therapy suspension reduces potential hemorrhage risk, exposing patient to thromboembolic risk. Similarly, drug dosage maintenance determines a greater probability of intra- and post-operative bleeding, safeguarding the patient from the formation of potential clots. In view of this, researchers are working to evaluate the possibility of not suspending or modifying anticoagulant therapy for minor surgical procedures, preventing bleeding complications by using hemostatic agents (fibrin glue, oxidized cellulose, topical antifibrinolytics) in the surgical site [5]. Some authors [2, 6, 7] highlighted a good hemostasis achievement (using simple or more structured local procedures) as goal for the management of both VKA and DOAC patients.

Hemocomponents for non-transfusional purposes present promising hemostatic potential among biomaterials [8]. Platelet-derived hemocomponents concentrate a hyper-physiological quantity of autologous platelets and related growth factors, directly in the surgical wound, with the aim to achieve a faster and more competent soft tissue healing [9].

As a matter of fact, these products may stimulate angiogenesis, enhance chemotaxis, and promote the rapid induction of connective tissue formation. L-PRF [10], acronym for fibrin rich in platelets and leukocytes, is one of the most used hemocomponents in outpatient surgery, due to its simple, quick, and standardized procedural protocol. L-PRF is obtained by centrifuging a blood sample without additives, with consequent separation of the clot in two distinct major phases: the plasma and erythrocyte components.

The rationale for choosing L-PRF as a hemostatic agent lies in its intrinsic property of facilitating blood clot formation through a rapid activation of the coagulation cascade. Furthermore, the formation of a physical barrier protecting the surgical site is not to be underestimated [11, 12].

Thanks to its three-dimensional architecture, L-PRF membranes offer greater biomechanical characteristics than other platelet concentrates [10]. This property facilitates membrane adaptation to different surgical sites, allowing its stabilization on soft tissues by compression or suturing. In addition to its resistance, the elasticity of this biomaterial is remarkable, allowing it to be slightly stretched without tearing.

The aim of this clinical observational study was to assess the efficacy of L-PRF as a hemostatic agent, comparing post-operative bleeding after single simple tooth extraction in patients under treatment with vitamin K antagonists (VKAs) or direct oral anticoagulants (DOACs).

## Material and methods

This study was designed in accordance with the principles expressed in the Helsinki Declaration (1964) and its later amendments (Fortaleza 2013), to Good Clinical Practice (GCP) guidelines, and it was approved by the Institutional Ethical Committee (CEUR Friuli Venezia Giulia, Italy) with univocal code 2016-OS022-ASUITS. The present study was recorded in a public register of clinical trials (Clinicaltrials.gov), with reference number NCT03124030.

The study was conducted in the Maxillofacial and Dental Department of the University Hospital of Trieste (Italy) and patients under oral anticoagulant therapy (VKA or DOACs) who needed a single tooth extraction were enrolled. Patients were selected on the basis of strict inclusion and exclusion criteria (Table 1) and then divided, according to their therapy, into group 1 (VKA) and group 2 (DOACs), respectively.

**Table 1:** Inclusion and exclusion criteria

Inclusion criteria	Exclusion criteria
Male and female patients ≥20 years	Smoke > 10 cigarettes per day
Healthy patients (≤ ASA 3)	Assumption of any antiplatelet medication and heparin medication or wash-out period after antiplatelet or heparin medication of at least one month.
At least two months of DOAC therapy with: dabigatran (PRADAXA) or rivaroxaban (XARELTO) or apixaban (ELIQUIS) or edoxaban (LIXIANA)	Assumption of OAT medications (warfarin, acenomarol) for patients in DOAC therapy
At least three months of OAT therapy with: warfarin (COUMADIN) or acenocumarol (SINTROM) for VKA group	Assumption of any DOACs, dabigatran (PRADAXA) or rivaroxaban (XARELTO) or apixaban (ELIQUIS) or edoxaban (LIXIANA), for patients in OAT therapy
Indication for extraction of a single tooth	Uncontrolled hypertension
No other contraindications for tooth extraction	Chronic hepatitis and/or reduction of hepatic function
Accepted platelet count within 30 days prior to the procedure must be > 50,000/dl.	Coagulopathy (in excess or defect)
INR measured the day of the procedure should be between 2.0 and 3.0 (VKA group)	Head and neck radiotherapy (previous 10 years).
	Extraction lasting more than 15 min, needing for osteotomy and/or flap elevation

During pre-operative evaluation, the study protocol was thoroughly explained to patients, who signed an informed consent form. Patients’ personal data and medical history were collected, with particular attention to the pharmacological therapy. Furthermore, the CHADS2-VASc and HAS-BLED scores were calculated in collaboration with an expert cardiologist (ADL), and the most recent blood serum exams were retrieved and reviewed. Other collected data are reported in supplementary materials (S1). Intraoral examination evaluated oral hygiene level and periodontal status by periodontal screening and recording (PSR).

**Fig. 1:**
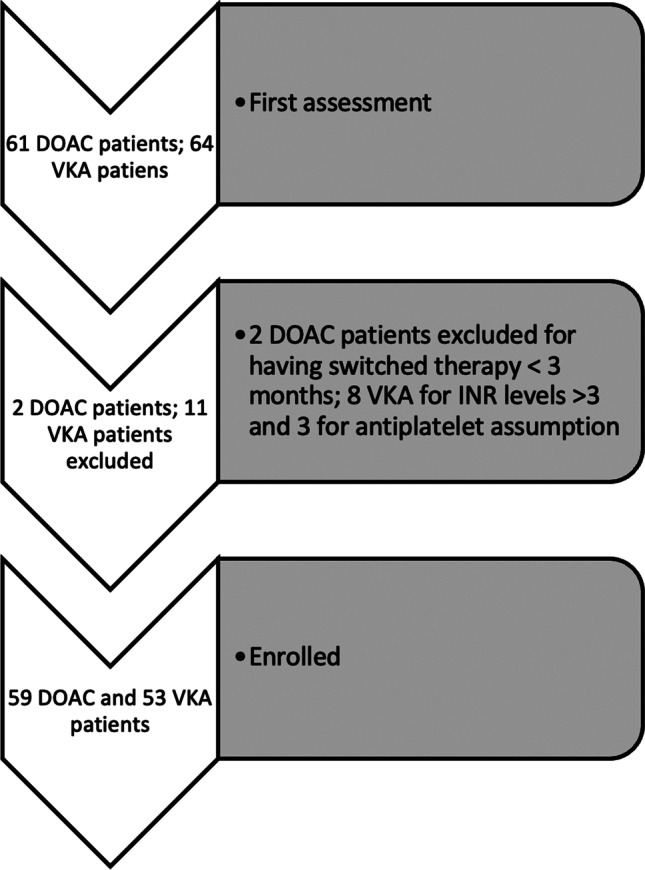
Enrolled and excluded patients

Intraoral radiographs and/or orthopantomographies were used to plan extractive therapy. When multiple extractions were indicated, priority was given to the symptomatic teeth. In asymptomatic patients, the most distal tooth programmed for extraction was selected. Each patient underwent professional oral hygiene one week before the extractive procedure (Fig. 1).

Blood pressure was recorded for both groups with CoaguChek XS (Roche Diagnostics GmbH, Mannheim, Germany), before tooth extraction; International Normal Ratio (INR) was registered for VKA group. Antibiotic prophylaxis was orally administered one hour before the extraction (Amoxicillin 2 gr or Clarithromycin 500 mg for allergic patients).

Subsequently, following cutaneous disinfection, blood withdrawal from cubital or cephalic vein of the non-dominant arm was performed using 9-ml blood collection tubes. The tube was immediately placed into a centrifuge (Intralock, Boca Raton, FL, USA) and operated for 18 min at 2700 rpm (as suggested by the producer for anticoagulated/antiaggregated/piastrinopenic patients). L-PRF was separated from the red blood component with scissors and then compacted using dedicated tools, obtaining a plug with a congenial shape for the alveolus.

The patient was asked to perform a one-minute mouth rinse with 0.2% chlorhexidine mouthwash before starting the surgical maneuvers. The time at which the procedure took place was recorded.

All the surgeries were performed by a single operator (FB) and only simple extractions were included (see Table 1 for exclusion criteria), i.e., carried out without elevation of mucoperiosteal flaps and ostectomies, in a maximum of 15 min (from periotomy to complete tooth extraction).

Local anesthesia (mepivacaine 20 mg/ml + adrenaline 1:100,000) was performed, and teeth were luxated and extracted with elevators and forceps. Following an accurate alveolar curettage, L-PRF plug was positioned and compacted inside the alveolus using appropriate tools and finally secured with non-absorbable 3/0 braided silk sutures. After aspirating the excess of saliva, a cotton roll was gently compressed over the surgical site for 20 s. The weight in grams of the cotton before and after imbibition was obtained with an analytical balance (AND HR-120 Max 120 g min 10 mg e = 1 mg day = 0.1 mg A&D INSTRUMENTS LTD, Oxfordshire, UK). All the intra-operative variables were collected as reported in supplementary materials (S2).

Within 30 min after surgery, patients began topical ice application keeping a gauze in compression. Patients were prescribed with chlorhexidine 0.2% 3 rinses/day (except for the first day) and paracetamol 1000 mg in case of pain, excluding any other NSAID. Once hemostasis was reached, the patient was discharged with a form to be filled with data related to pain-VAS (score 0 to 10), paracetamol intake, any bleeding event and its management.

The seventh day sutures were removed and any biological complication (ecchymosis, hematoma, swelling, infection, nerve injury) was recorded, as well as the presence of phlebitis, bruising, or hematomas at the sampling site on the arm. Bleeding events have been classified using Iwabuchi classification (reported in supplementary materials as S3) [13, 14].

Bleeding score of 3 or greater was considered clinically significant and classified as a relevant post-operative bleeding complication. In addition, the onset, course, and severity of complications, as well as the procedures undertaken by the patient or the operator to solve them, were registered.

### Statistical analysis

The data were processed using SPSS software version 15.0 for Mac OS X (SPSS® Inc., Chicago, Illinois, USA). After testing the normality of the data using Shapiro-Wilk test, and the equality of variance between the datasets using Levene test, parametric methods for continuous variables were chosen.

Differences between groups in regard to gender and age distribution were reported. The differences between VKA and DOACs group regarding comorbidities, previous post-surgical bleeding episodes, associated coagulopathy, ACE inhibitor intake, smoking habits, last referral dental visit, were reported. CHA2DS2-VASC and HAS-BLED values and the time of assumption of the anticoagulant were reported. Difference between groups in platelet count, ALT, AST, γ-GT, creatinine, hematocrit, and hemoglobin prior to oral surgery were reported. The differences between the two groups in pre-operative systolic and diastolic pressure and the amount of cotton imbibition were also reported. The difference in bleeding episodes (yes or no) was analyzed using Chi-square test, while the amount of bleeding according to Iwabuchi [13] was compared with Mann-Whitney test. Pain-VAS daily values and paracetamol intake were analyzed with ANOVA for repeated measures. For the comparison of the cotton roll imbibition the *T* test for independent samples was used.

In order to evaluate the variables implicated in the bleeding events, a multivariate regression analysis with stepwise insertion method was conduct comparing the bleeding events with age, gender, VKA, DOAC, degree of gingival inflammation, granulation tissue, and multi-single-rooted elements. Results are reported in Table 5.

A value of *p* <0.05 was chosen as the level of statistical significance.

## Results

A total of 112 patients under oral anticoagulation therapy (59 patients for DOAC and 53 for VKA group) needing tooth extraction were enrolled in this study between September 2018 and January 2020 (Fig. 1).

### Pre-operative variables (T0)

The two groups were homogeneous for all parameters, evenly distributed by gender (*p* = 0.93) and age (*p* = 0.79), with a mean of 76.6 ± 9.2 years for VKA and 76.8 ±10.4 for DOAC, as well as by pathology for which they were under anticoagulant therapy.

In the DOAC group, 15 patients (25.4%) took dabigatran, 17 (27.1%) rivaroxaban, 17 (28.8%) apixaban, and 11 (18.6%) edoxaban, while in the VKA group 51 (96.2%) took warfarin and the remaining 2 (3.8%) acenocoumarol. The most frequent indication both for DOACs and VKA was atrial fibrillation, with 39 (73.6%) and 39 (66.1%) cases, respectively.

Patients were equally distributed with regard to the time elapse from the last dental visit (Table 2).

**Table 2:** Comparison between VKA and DOAC groups at baseline (T0)

		VKA (*n*=53)	DOAC (*n*=59)
Age (mean ± SD)		77.42 ± 10.75	76.85 ± 11.58
Gender (*n*,%)	Men	31 (58.5%)	35 (59.3%)
	Women	22 (41.5%)	24 (40.7%)
Drug therapy (*n*,%)	Dabigatran	-	15 (25.4%)
	Apixaban	-	16 (27.1%)
	Rivaroxaban	-	17 (28.8%)
	Edoxaban	-	11 (18.6%)
	Warfarin	51 (96.2%)	-
	Acenocumarol	2 (3.8%)	-
Related pathology (*n*,%)	Atrial fibrillation	39 (73.6%)	39 (66.1%)
	Deep venous thrombosis	4 (7.5%)	3 (5.1%)
	Ictus/TIA	2 (3.8%)	5 (8.5%)
	Pulmonary embolism	2 (3.8%)	4 (6.8%)
	Combined	6 (11,3%)	8 (13.5%)
Time of assumption (*n*,%)	< 6 mo	1 (1.9%)	10 (17%)
	< 1 y	5 (9.4%)	6 (10.2%)
	≥ 1 y	47 (88,.7%)	43 (72.9%)
Reported post-surgical hemorrhages (*n*,%)	Yes	3 (6.7%)	6 (10.2%)
	No	50 (94.3%)	53 (89.8%)
ACE-inhibitors assumption (*n*, SD)	Yes	10 (18,.9%)	19 (32.2%)
	No	43 (81.1%)	40 (67.8%)
PPI assumption (*n*,%)	Yes	15 (28.3%)	18 (30.5%)
	No	38 (71.7%)	41 (69.5%)
Smoking habit (*n*,%)	Yes	6 (11.31%)	2 (3.3%)
	No	47 (88.7%)	57 (96.6%)
CHA2DS2-VASC (mean±sd )	Score (from 0 to 9)	4.66±1.39	4.44±1.54
HAS-BLED (mean±sd)	Score (from 0 to 9)	1.90±0.86	2.04±0.65
Previous VKA therapy (*n*,%)	Yes	-	35 (59.3%)
	No	-	24 (40.7%)
Last dental visit (*n*,%)	< 1 year	53 (100%)	58 (98.3%)
	≥ 1 year	0 (0 %)	1 (1.7%)

In patients receiving VKA, mean CHA2DS2-VASC and HAS-BLED scores were 4.66±1.39 and 1.90±0.86, respectively. In subjects treated with DOAC, mean CHA2DS2-VASC score was 4.44±1.54, while mean HAS-BLED score was 2.04±0.65). The two groups resulted of augmented stroke risk (*x*>0 male; *x*>1 female, as female gender represents per se a risk factor bringing 1 point to the score) but not of augmented bleeding risk (both mean values below 3, as cut off) (Table 2).

Hematologic parameters (sampled within 30 days before intervention) were found in the normal range in all patients [number of platelets, hepatic enzymes (ALT U/L, AST U/L, and γ-GT), serum creatinine, hematocrit, and hemoglobin]. Detailed descriptive data are listed in supplementary materials as S4.

### Intra-operative variables (T1)

Blood pressure and INR were measured in all subjects just before proceeding with the surgical *maneuvres*. Regarding this last parameter, average INR value was 2.4±0.43 in VKA group. Mean systolic 130±15.68 mmHg and diastolic 74±11.88 mmHg for VKA group and 134±16.33 mmHg and 76±10.06 mmHg for DOACs group.

Indication for extraction and type of dental element were equally distributed in the groups. The most frequent indication for tooth extraction was endodontic/conservative [31 (52.5%) and 28 (53%) patients for DOAC and VKA groups, respectively], with no significantly different distribution along with the other indications (i.e*.*, periodontal issues and combination of the above).

There were no significant differences between the two groups regarding gingival inflammation of the surgical site (*p*=0.08) and amount of granulation tissue of the post-extraction socket (*p*=0.90) (Table 3).

**Table 3:** Comparison between VKA and DOAC at T1

		VKA (*n*=53)	DOAC (*n*=59)	*p*-value
Tooth (*n*,%)	Single-rooted	23 (43.4%)	30 (50.9%)	0.43
	Multi-rooted	30 (56.6%)	29 (49.1%)	
Indication for extraction (*n*,%)	Periodontal disease	9 (17%)	12 (20.3%)	
	Decay	28 (53%)	31 (52.5%)	0.97
	Pericoronaritis and apical involvement	1 (1.9%)	1 (1.7%)	
	Combined	15 (28.3%)	15 (25.4%)	
Blood pressure (mean, SD)	Systolic	130 (15.68)	134 (16.33)	0.13
	Diastolic	74 (11.88)	76 (10.06)	0.45
Surgical time (*n*,%)	< 10 min	43 (81.1%)	52 (88.1%)	-
	≥10 min	10 (18.9%)	7 (11.9%)	

### Immediately post-operative assessment (T2)

Most of the patients achieved complete hemostasis immediately, with an average cotton roll imbibition rate of 0.03±0.02 g and 0.04±0.04 g for VKA and DOAC, respectively. No significant differences between the two groups were demonstrated for this item (*p*=0.65).

### Late post-operative assessment (T3)

Post-operative bleeding was recorded in nine patients (17%) for VKA group and nine patients (15.3%) for DOACs group. No significant difference has been registered (*p*=0.31).

In particular, bleeding was distributed as follows in VKA patients: bleeding was managed with simple compression (once or twice a week) in seven patients (13.2%), while in two patients (3.8%) bleeding was stopped with simple compression (more than twice a week).

In DOAC group, all the nine patients reported bleeding stopped with simple compression (once or twice a week). None of the patients of the two groups needed a medical support for managing of bleeding (score 4 according to the classification used).

Seven days after surgery, no cases of post-extractive complications, such as ecchymoses, hematoma, swelling, neurological lesions, and or surgical site infections requiring antibiotic therapy occurred (Table 4).

**Table 4:** comparison between VKA and DOAC at T3

	*N and %*	VKA (n=53)	DOAC (n=59)	p-value
Taste of blood	Yes	7 (13.2%)	1 (1.7%)	0.02
	No	46 (86.8%)	58 (98.3%)	
Bleeding	Yes	9 (17%)	9 (15.3%)	
	No	44 (83%)	50 (84.7%)	
Bleeding events according to Iwabuchi	No bleeding	44 (83%)	50 (84.7%)	
	One compression with gauze	7 (13.2%)	9 (15.3%)	
	More than two compressions with gauze	2 (3.8%)	0 (0%)	0.31
	Need of pharmacological intervention	0 (0%)	0 (0%)	
	Need of surgical intervention	0 (0%)	0 (0%)	
Post-surgical complications	Hematoma, swelling, infection, nerve injury, ecchymosis	0 (0%)	0 (0%)	-
	Hyperplastic clot	0 (0%)	2 (3.4%)	

Among the DOACs, hyperplastic clots were detected in two patients (3.4%). In the first case, the clot was confined to the alveolus and managed by revision of the surgical site. In the second patient, however, the size of the clot was considerable (about 30mm), and its traumatic rupture with consequent hemorrhage on the seventh day had anticipated by a few hours the removal of the suture. L-PRF was not repositioned following alveolar revision while, in the second case, a tranexamic acid-soaked gauze was used to facilitate hemostasis.

The sensation of blood taste during the seven post-operative days was significantly more frequent in VKA group (*p* = 0.018). No differences between the two groups emerged with respect to the intake (yes/no) of paracetamol in the 7-day post-operative period (*p* = 0.5). No differences in the amount of paracetamol (mg) has been observed between groups at each time-point.

Similarly no differences in pain-VAS values have been observed between the two groups on post-operative days (*p*=0.409).

Apparently, bleeding events occurred more frequently in those patients with relevant amount of granulation tissue (described as severe) and gingival inflammation (described as severe).

Finally, 20 patients (7 VKA and 13 DOAC) reported ecchymoses in the site of blood sampling (forearm).

Multivariate regression analysis failed to highlight any correlation to the examined variables but with presence of gingival inflammation (Table 5).

**Table 5:** Multivariate regression analysis with stepwise insertion method to compare bleeding events with age, gender, VKA, DOAC, degree of gingival inflammation, granulation tissue, and multi-single-rooted elements. Gingival inflammation, registered as absent, mild, present, and severe, was grouped in dichotomic way (absent and mild, as absent and present, and severe as present) for statistical purposes

	Coeff	[95% CI]	*p-value*
Age	−0.005	−0.011 to 0.002	0.14
Gender			
Male	1		
Female	−0.005	−0.145 to 0.135	0.95
Therapy			
DOAC	1		
VKA	−0.028	−0.165 to 0.109	0.69
Root			
Multi-rooted tooth	1		
Single-rooted tooth	0.033	−0.104 to 0.171	0.63
Gingival inflammation			
Presence	1		
Absence	0.223	0.033 to 0.414	0.02
Granulation tissue			
Presence	1		
Absence	−0.054	−0.247 to 0.139	0.58

## Discussion

This observational longitudinal study aimed to evaluate the hemostatic effect of L-PRF plugs in post-extractive sites in anticoagulated patients (VKA and DOAC assumption) with a not-ceasing protocol of the drug. Previous experience of our research group, together with the growing body of literature, highlighted how the not-ceasing protocol can be adopted for minor oral surgeries without the risk of serious hemorrhagic complications [5, 14, 15]. However, the complete elimination of clinically relevant bleeding episodes has not been previously reported in such population. For example, a recent study conducted on 232 elderly patients reported 39 post-operative bleeding events (16%), with an augmented incidence for rivaroxaban users [16]. Also in the previously reported study of the authors of the present [14], rivaroxaban was the most associated to bleeding events, followed by apixaban, dabigatran, and edoxaban although not significant. Limited bleeding occurrence in anticoagulated patients was reported by a recent meta-analysis, with significantly greater incidence than in healthy patients [17]. In the present study, the global incidence among the treated population was 16% using L-PRF as local hemostatic agent, equally distributed in DOAC and VKA groups, in perfect accordance with the results reported by Inokoshi and coworkers. However, reported bleeding events can be considered as mild or clinically irrelevant (without the need of medical intervention). A comparison can be made with the two anticoagulated cohorts without the use of L-PRF of our previous work [14], given its identical protocol (excluding L-PRF). The bleeding events between the four cohorts appear similar not reaching significant difference, even if some serious bleeding events occurred in the NO-PRF cohorts (3 out of 130), with necessity of medical assistance. It should be noted that baseline bleeding risk assessed with HAS-BLED scoring test was significantly higher in the cohorts of the present study, when compared to our previous work [14]. This potential bias might occur against the observed difference between groups, which could even be more relevant. An interesting correlation emerged between bleeding events and gingival inflammation. This positive correlation confirmed a well-known phenomenon: inflamed tissues are more prone to post-operative bleeding than healthy ones [18, 19], underlying the crucial role of gingival inflammation control and periodontal preparation before oral surgery interventions, especially in patients under anticoagulation therapy. Conversely, no differences in bleeding events have been observed for the other analyzed variables such as the different types of tooth (single or multi-rooted) for both groups (Table 3). Similarly, a study conducted on 1.833 eligible patients failed to demonstrate a correlation between bleeding complications and localization of the surgical procedures (jaws, anterior, or posterior teeth) [20].

Despite post-extractive bleeding does not represent a life-threating condition, it can impact on post-extractive morbidity, patient psychology, and in some isolated cases on patient health. A retrospective study analyzing more than 1800 oral surgical procedures and following immediate or delayed bleeding revealed that uncomplicated extractions were the main cause of post-operative bleeding. Therefore, single tooth extraction should be not underestimated even in comparison with more invasive oral surgery procedures. In fact, post-operative management plays a fundamental role, mostly in secondary intention wound. For example, the correct placement of cotton swab, hemostatic agents, and adequate time of compression is crucial [20].

Many other hemostatic agents have been described in literature, such as porcine collagen, tranexamic mouthwash, resorbable oxycellulose dressing, fibrine adhesive, calcium sulfate, chitosan, and epsilon-aminocaproic acid [21–28]. Moreover, also platelet concentrates have been tested with the same purpose [29–31]: a recent case series [32] reported good clinical outcomes on the use of PRF in DOAC patients, recording no bleeding events in rivaroxaban and apixaban users. A recent systematic review including three studies on this topic, highlighted that PRF use did not significantly decrease bleeding events incidence and, likewise with our results, pain-VAS analysis showed that treatment with PRF did not significantly improve mean post-operative pain scores [30]. However, the aforementioned review included three studies conducted on nonhomogeneous patients: as antiplatelet patients were compared with anticoagulated patients, different pharmacodynamics of these drugs may have been biased the results.

As the preparation of platelet derivatives includes the withdraw of a venous blood sample, also local forearm complications such as bruising or hematoma should be taken into account. In the present study, 20 patients (7, 13.2% of VKA and 13, 22.0% of DOAC) reported ecchymoses in the site of blood sampling, with spontaneous resolution. This aspect should be taken into account in cost and benefit evaluation for the use of L-PRF as hemostatic agent in anticoagulated patient. Luckily, venipuncture-induced nerve injuries are rare; factors other than direct nerve contact appear necessary for chronic pain syndrome to occur [33]. A post-donation interview of 1000 blood donors showed that 22.7% of them had bruise and 1.7% had hematomas. Bruise and hematoma were significantly more frequent in women than in men (30.6% vs. 13.2% and 2.9% vs. 0.4%, respectively) [34].

The present data highlight a similar prevalence of post-operative bleeding among patients treated with L-PRF and those not of other reports. Nonetheless, type of post-operative bleeding look different: according to Iwabuki classification, if cohorts treated without the use of L-PRF of our previous report are considered [14], L-PRF groups did not report clinically relevant bleeding needing medical intervention at all.

In the first structured report of L-PRF used in anticoagulated patients published by Sammartino and coworkers in 2011 [11], among 168 post-extraction sockets only two patients reported hemorrhagic complications (4%), all of which resolved a few hours after the surgery by compression and hemostatic topical agents. Ten patients (20%) showed mild bleeding, which spontaneously resolved or was resolved by minimal compression less than 2 h after surgery. No case of delayed bleeding was reported. The remaining 38 patients (76%) showed an adequate hemostasis after dental extraction. The authors concluded that similar hemostatic outcome can be reached with other techniques such as oxidate cellulose mesh or tranexamic acid. The main advantage of using PRF is not only related to its antihemorrhagic properties; PRF is also a filling healing biomaterial accelerating wound closure. Its immune content may help to protect the extraction sockets against unavoidable infections, and by enhancing soft tissue healing. Furthermore, PRF reduces the duration of the contamination of the surgical site by oral bacteria. None of PRF patients of this study reported alveolitis, while two patients showed hyperplastic clots at suture removal, 7 days post-surgery. In NO-PRF cohorts of the former study of the author, only two post-extractive alveolitis out of 130 extraction were recorded, both in the VKA and DOAC groups. During PRF centrifugation, platelets are activated, and cytokines are released. Analysis of the PDGF-BB, TGF-b1, and IGF-I within the PRF clot serum revealed that slow fibrin polymerization during PRF processing leads to the intrinsic incorporation of platelet and cytokines, which allows for their progressive release over time (7 to 11 days), as the mesh of fibrin disintegrates [35]. Moreover, during PRF processing, leucocytes could also secrete cytokines in reaction to the hemostatic and inflammatory phenomena induced by the centrifugation [36]. This phenomenon may explain the absence of post-operative infections when PRF is used (against two alveolitis out of 130 extractions in the NO-PRF cohorts).

The present report shows relevant criticisms that can be ascribed firstly to the strict inclusion criteria for patient enrollment, with the purpose of lower as much variables as possible (only single simple tooth extraction), that inevitably brought to a limited number of eligible patients, therefore reducing the power of the statistical analysis. Moreover, the outcomes of the present study cannot be extended to other surgical procedures. Furthermore, the non-experimental design of the study is not capable of giving us back the real impact of L-PRF on the prevalence of post-operative bleeding after tooth extraction on anticoagulated patients, lacking a control group. Randomized controlled trials are needed to better understand the role of this biological filler/wound dressing.

## Conclusions

The use of L-PRF was able to bring the patients only to limited mild late post-operative bleedings without the need of medical intervention. However, the prevalence of bleeding events is not dramatically lowered if compared with previous report without the use of L-PRF. The reduced number of patients and the limited amount of bleeding complications should be taken into account. Further studies are needed to better estimate the role of this platelet derivative.

## Supplementary information

ESM 1
