# Supplementary material for: Is L-PRF an effective hemostatic agent in single tooth extractions? A cohort study on VKA and DOAC patients
Source: Clin Oral Investig. 2023 Jan 28;27(6):2865–74. doi: 10.1007/s00784-023-04880-z (PMC10264503; doi:10.1007/s00784-023-04880-z)
Supplement: Supplementary file 1 — ESM 1 [file 784_2023_4880_MOESM1_ESM.docx]

Table 1S. Preclinical assessment S1

| Personal data | Gender. age |
| --- | --- |
| General evaluation | Medical history |
| Pharmacological therapy | The concomitant use of antiplatelet agents. or drugs that have known interactions with anticoagulant therapy. ACE inhibitors and pump inhibitors |
| Smoking habits | y/n. how many |
| Anticoagulant therapy | Type. duration. prescription |
| Previous post-surgical bleeding | Referred by the patient |
| Coagulation disorders | Depending on liver. platelets. vessels. other interfering drugs |
| Blood sample | PT-INR (VKA group). platelet count. Cr. hepatic function. Ht. Hb |
| Tooth extraction indication | Endodontic. periodontal. etc. |

Table 2S. Intraoperative variables S2

| Preoperative fast PT-INR record | *For VKA therapy the value must be between 2.0 and 3.0* |
| --- | --- |
| Site of extraction | *According to ADA system* |
| Gingival inflammation of the extraction site | *None to mild. moderate to severe* |
| Starting time of intervention | *From the periotomy* |
| Finishing time of intervention | *When the tooth is completely extracted* |
| Granulation tissue in the socket | *None to scarce. moderate to abundant* |
| Roll cotton imbibition assay | *Before/after weight comparison* |

Table 3S. Bleeding classification according to Iwabuchi S3

| CODE | CLINICAL EVIDENCE |
| --- | --- |
| 0 | no bleeding |
| 1 | bleeding stopped by simple compression (once or twice a week) |
| 2 | bleeding stopped by simple compression (more than twice in the week) |
| 3 | bleeding requiring pharmacological intervention (tranexamic acid) |
| 4 | bleeding that requires medical intervention (surgery with additional sutures and / or diathermal-coagulation) |

Table 4S. blood tests at baseline (T0) S4

| Blood test | VKA (n=53) mean *± sd* | DOAC (n=59) mean *± sd* |
| --- | --- | --- |
| Platelet x10^3/µL | 218 ± 64.27 | 214 ± 57.68 |
| ALT U/L | 19 ± 9.13 | 18 ± 9.96 |
| AST U/L | 23 ± 6.86 | 22 ± 9.09 |
| γ GT U/L | 31± 23.87 | 30 ± 24.95 |
| Creatinine mg/dl | 1 ± 0.31 | 1 ± 0.48 |
| Ht % | 43 ± 7.67 | 41 ± 4.57 |
| Hb g/dl | 13 ± 1.74 | 13 ± 1.69 |
